# Epidemiology and clinical features of PFAPA: a retrospective cohort study of 336 patients in western Sweden

**DOI:** 10.1186/s12969-022-00737-z

**Published:** 2022-09-15

**Authors:** Karin Rydenman, Hanna Fjeld, Josefine Hätting, Stefan Berg, Anders Fasth, Per Wekell

**Affiliations:** 1 Department of Pediatrics, NU Hospital Group, Uddevalla, Sweden; 2 Department of Pediatrics, Institute of Clinical Sciences, University of Gothenburg, Gothenburg, Sweden; 3 Department of Medical Internship, NU Hospital Group, Trollhättan, Sweden; 4 Department of Pediatrics, Skaraborg Hospital, Lidköping, Sweden; 5 Department of Pediatric Rheumatology and Immunology, Queen Silvia Children’s Hospital, Gothenburg, Sweden

**Keywords:** PFAPA, Periodic fever, Autoinflammatory disease, Incidence

## Abstract

**Background:**

Periodic fever, aphthous stomatitis, pharyngitis and cervical adenitis (PFAPA) syndrome is generally regarded as the most common autoinflammatory disease, but the epidemiology of the disease is largely unknown. The objectives of this study were to estimate the annual incidence and describe the clinical features of PFAPA in a large cohort from western Sweden.

**Methods:**

The study retrospectively included children < 18 years of age diagnosed with PFAPA between 2006 and 2017 at three hospitals: NU Hospital Group, Skaraborg Hospital and Queen Silvia Children’s Hospital. Patients were identified by searching for relevant diagnostic ICD-10 codes in the comprehensive electronic medical records and data were retrieved by reviewing case records. To estimate incidence, patients with symptom onset from January 1, 2006, to December 31, 2016, were included. Population data for the study area during this period were retrieved from Statistics Sweden.

**Results:**

In this study, 336 patients with PFAPA were identified. Of these, 156 (46%) were girls and 180 (54%) were boys. Almost 90% of the children with PFAPA (291 patients) experienced their first symptoms before the age of 5 years and fewer than 3% presented at ages above 10 years. Pharyngitis was the most common symptom during febrile episodes, followed by cervical adenitis and aphthous stomatitis. Fourteen percent of the patients displayed atypical features, of which skin rash was the most common.

To calculate incidence, 251 patients with symptom onset during the study period were identified. The mean annual incidence was estimated at 0.86/10,000 for children < 18 years of age and 2.6/10,000 for children < 5 years of age.

**Conclusions:**

This study adds to the understanding of the epidemiology of PFAPA syndrome by presenting incidence rates based on a large cohort and in different age groups in a population-based setting. It also shows the distribution of age of onset of PFAPA, with a peak in 1-year-olds and waning at older ages. Signs and symptoms of PFAPA syndrome were similar in children with symptom onset before vs. after 5 years of age.

## Background

Periodic fever, aphthous stomatitis, pharyngitis and cervical adenitis (PFAPA) syndrome was first described as a clinical entity in the late 1980s by Marshall et al. [1, 2]. They identified a group of patients, all children and most of them with symptom onset before the age of 5 years, with regularly recurring fever episodes accompanied by aphthous stomatitis, pharyngitis and cervical adenitis in the absence of upper respiratory tract infection. After this first report, PFAPA syndrome has been increasingly recognized and characterized with reports of patient cohorts from many parts of the world. The regularity of the fever episodes, the stereotypical features of every episode, the marked elevation of inflammatory markers during the episodes and normalization in the asymptomatic intervals remain hallmarks of the syndrome [3]. The clinical picture of periodic fevers, as well as studies of pathogenesis showing the activation of the innate immune system in PFAPA [4–6] has placed the syndrome among the autoinflammatory disorders, even though the exact etiology of the syndrome remains enigmatic [7]. There appears to be a genetic predisposition to develop PFAPA syndrome, but no single responsible gene has been found and the syndrome is probably inherited as a complex genetic disease [8], possibly with environmental triggers.

Diagnostic criteria for PFAPA syndrome were first proposed by Marshall et al. [2] and later modified by Thomas et al. [9], building on their clinical observations. The latter set of criteria, often referred to as the modified Marshall criteria, have been widely used, but, as experience of PFAPA syndrome has accumulated and as the field of autoinflammatory disorders has expanded dramatically since they were published, it has become evident that they have limitations. The need for new, evidence-based criteria for PFAPA syndrome has been recognized and new classification criteria were recently proposed [10], but, as yet, they have not been independently validated. The new classification criteria were developed to enable the creation of homogeneous patient groups in a research context and not for clinical practice.

The modified Marshall criteria state that symptom onset should occur before 5 years of age, but even though this is often the case, it is now widely recognized that it may start later in life, even in adulthood [11–14]. In addition, several rare monogenic autoinflammatory disorders (mAID) have been defined, some of which may display clinical features that overlap with PFAPA [15, 16]. The most important clinical characteristics for distinguishing PFAPA from other disorders are the presence of regular, stereotypical and self-limiting fever episodes, accompanied by signs and symptoms from the oro-pharyngeal area. Some clinical features have been highlighted as being atypical of PFAPA, including skin rash, arthritis, severe abdominal pain, conjunctivitis, periorbital edema, diarrhea, chest pain, episodes triggered by cold or exercise and length of fever episodes ≥7 days. In patients who display these features, other etiologies, including rare mAIDs, should be considered [7, 17]. The presence of these atypical signs and symptoms does not, however, per se rule out PFAPA according to the current criteria [9, 10].

Even though there is still some controversy regarding the diagnostic criteria, PFAPA syndrome is well recognized as a clinical entity and is generally regarded as the most common autoinflammatory disease. Population-based studies of the disease remain scarce, together with reliable data on the frequency of the syndrome. The prevalence of PFAPA is difficult to determine, as the course of the syndrome may fluctuate, and although it is usually self-limiting with the resolution of fever episodes after some years, in some patients the periodic fevers recur even after long periods of remission. Few studies have reported data on the incidence of PFAPA. Forsvoll et al. calculated an annual incidence of 2.3/10,000 in children up to 5 years of age, based on 34 children seen at one university hospital in Norway [18]. A recent review article by Renko et al. suggested similar numbers, with an annual incidence in Finland of 2/10,000 in children < 5 years of age, an estimation based on a previously published cohort consisting of 133 patients with periodic fevers who had undergone tonsillectomy and of whom at least 34 did not fulfill the modified Marshall criteria, as fever was the only symptom during episodes [19, 20]. Ng et al. presented an annual incidence rate of 0.098/10,000 in children aged 0–16 years, based on 51 patients from their tertiary clinic, with an increase to 0.247/10,000 during the COVID-19 pandemic [21].

The objective of this study was to further the understanding of the epidemiology of PFAPA by investigating a large cohort of patients with PFAPA in Region Västra Götaland, Sweden. The specific aims of the study were to: 1) Describe the clinical characteristics of the syndrome in a large cohort, including the distribution of age of onset of PFAPA in childhood, signs and symptoms associated with the fever episodes, laboratory investigations, treatment and a family history of recurrent fever and tonsillectomy; 2) Analyze the occurrence of atypical symptoms in patients with PFAPA syndrome onset before and after 5 years of age and 3) Estimate the annual incidence of PFAPA in children up to 18 years of age.

## Methods

### Study area and population

**Fig. 1:**
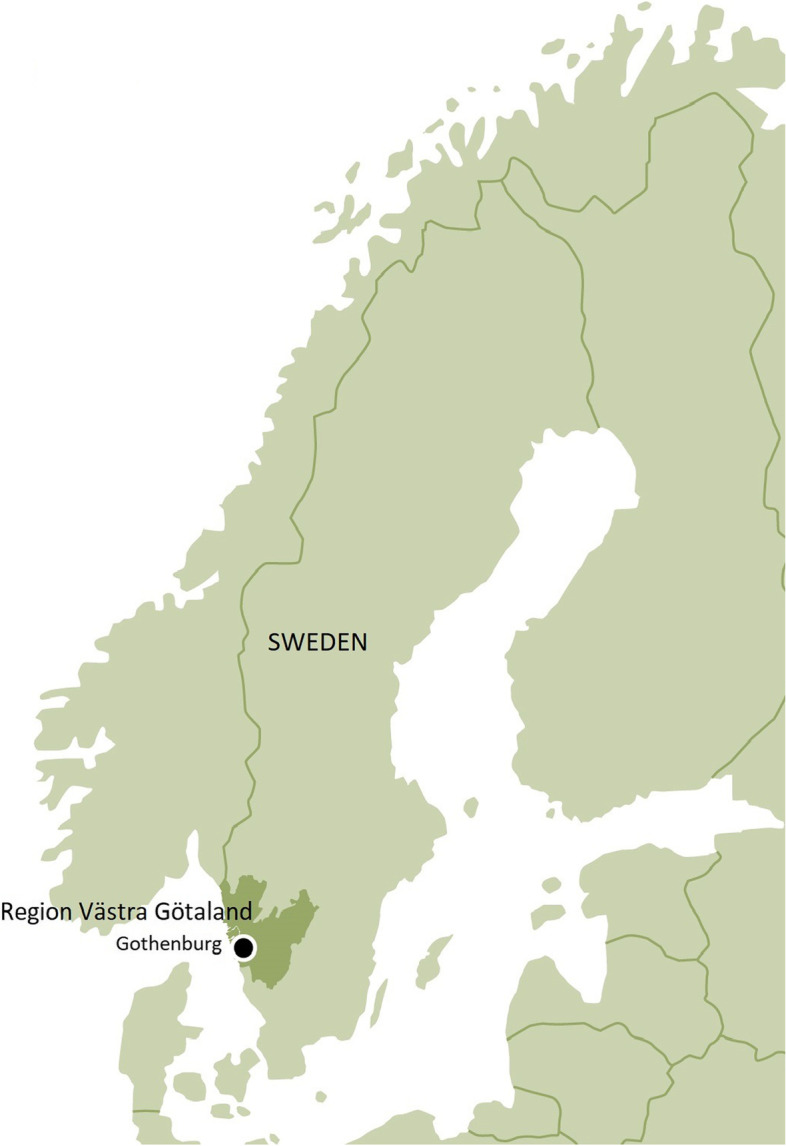
Map showing the geographical area of the study

In Sweden, all children up to the age of 18 years have access to public healthcare free of charge. The study was conducted in Region Västra Götaland, a region with mixed rural and urban areas, including Gothenburg, Sweden’s second largest city, as well as several smaller cities (Fig. 1). Three of four hospital organizations with pediatric departments in Region Västra Götaland were included in this study. Each hospital functions as a referral center for children with rheumatic and autoinflammatory disorders in a defined geographical area. The three hospital organizations included in the study were: NU Hospital Group, situated in Trollhättan and Uddevalla; Skaraborg Hospital, situated in Lidköping and Skövde; and Queen Silvia Children’s Hospital in Gothenburg. NU Hospital Group and Skaraborg Hospital provide pediatric care at secondary level, while Queen Silvia Children’s Hospital is the only pediatric university hospital in the region and serves patients resident in the Gothenburg area at secondary level, as well as all pediatric patients in Region Västra Götaland at tertiary level. The geographical area served by the three hospitals constitutes the catchment area of the study. The fourth hospital organization in Region Västra Götaland, Södra Älvsborg Hospital, was not included in the study. There are also several primary and a few additional outpatient secondary care facilities in the region, where children can receive healthcare. However, these facilities are mainly dedicated to general pediatrics, rarely diagnose and treat patients with autoinflammatory disorders including PFAPA and were not included in the study.

Data on the population within the catchment area were retrieved from Statistics Sweden [22]. At the beginning of the study period in 2006, the area had a population of 1,257,995, including 263,745 (21%) children below the age of 18 years, of which 49% were females and 51% males. At the end of the study period in 2016, the total population was 1,370,723, of which 280,022 (20%) were children and the gender distribution among them was 48% female and 52% male. The corresponding figures for children < 5 years of age was 70,083 in 2006 and 82,139 in 2016 (49% female/51% male). The population in the study area comprised 14% of the total Swedish population.

### Data collection

Children < 18 years of age diagnosed with PFAPA at any of the study hospitals during the period January 1, 2006 - October 31, 2017, were included retrospectively. A few patients were referred to the hospitals in the study even though they resided outside the catchment area. Place of residency was checked and only patients resident in the catchment area at the time of diagnosis were included when incidence rates were calculated.

**Fig. 2:**
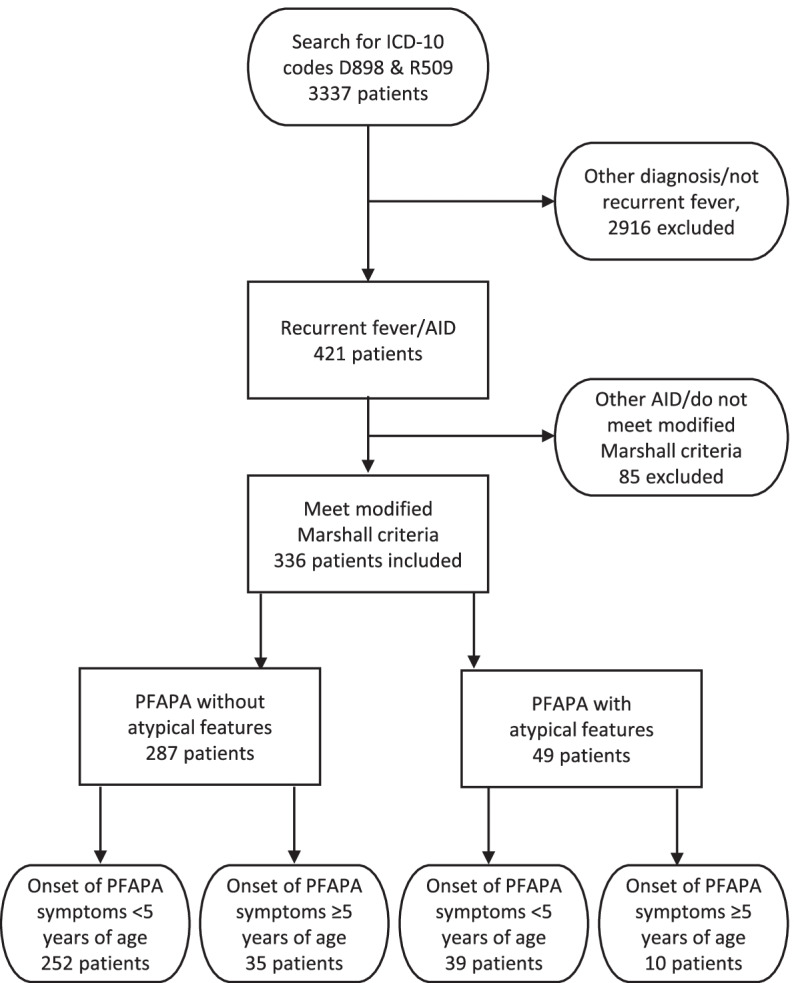
Flow chart of the search results, excluded cases and subgroups among included patients

As there are currently no universally accepted diagnostic criteria for PFAPA, we defined inclusion criteria based on the modified Marshall criteria [9], but, as there is now consensus that the onset of PFAPA can occur later in life, we included all children up to 18 years of age (Table 1). Potentially eligible patients were identified by searching for the ICD-10 code D898 in the comprehensive electronic medical records (Melior, Cerner Sweden). D898 is the ICD-10 code used for PFAPA by the pediatric units in the study area, in accordance with recommendations from the Swedish National Board of Health and Welfare (personal correspondence, KR). To ensure that no eligible patients were missed, an additional search for the ICD-10 code R509, which is the symptom code for fever without any specific diagnosis, was performed among patients registered as non-emergency outpatients. The medical records of all the patients identified by these search criteria were reviewed to identify patients with PFAPA. Patients with other diagnosed etiologies of the fever, such as infections, autoinflammatory conditions other than PFAPA or autoimmune conditions, or who had recurrent fevers but did not meet the modified Marshall criteria were excluded. The medical records of the remaining patients were further scrutinized by two of the authors (KR, PW). Patients who fulfilled the inclusion criteria and were not suspected of having another AID, based on expert opinion guided by the clinical classification criteria for monogenic AID [10], were included (Fig. 2).

**Table 1:** Inclusion criteria and definitions

PFAPA without atypical features:	PFAPA with atypical features:
• Meet modified Marshall criteria [9]: I. Regularly recurring fevers with an early age of onset (< 5 years of age) II. Constitutional symptoms in the absence of upper respiratory infection with ≥ 1 of the following clinical signs: a). Aphthous stomatitis, b) Cervical lymphadenitis, c) Pharyngitis III. Exclusion of cyclic neutropenia IV. Completely asymptomatic interval between episodes V. Normal growth and development N.B. In clinical practice, cyclic neutropenia was often excluded on clinical grounds. and: • Duration of episodes < 7 days • No atypical features	Meet modified Marshall criteria and have at least one atypical feature, i.e. • length of fever episodes ≥ 7 days • diarrhea • chest pain • skin rash • arthritis • severe abdominal pain • sensorineural hearing impairment • conjunctivitis • periorbital edema • cold- or exercise-triggered episodes and not suspected of having another AID, based on expert opinion guided by the clinical classification criteria for monogenic AID [10]
**PFAPA without atypical features but with late onset:**	**PFAPA with atypical features and with late onset:**
Meet all the criteria stated above, except that the onset of symptoms occurred at ≥ 5 years of age	As stated above but with symptom onset at ≥ 5 years of age

For the included patients, data were retrieved from the medical records and entered into a structured protocol. The protocol included the following information: time of disease onset, time of PFAPA diagnosis, symptoms during and between fever episodes, clinical signs, laboratory tests, treatment and a family history of hereditary disorders including inflammatory conditions. Separate subgroups for children with symptom onset before or after 5 years of age were created to enable comparisons with previous studies. In addition to the stereotypical PFAPA features of aphthous stomatitis, pharyngitis and cervical lymphadenitis, atypical symptoms were identified in the medical records of some patients (Table 1). Abdominal pain, nausea, vomiting, myalgia, arthralgia or headache are common in PFAPA and these symptoms were not labeled atypical.

### Statistical analyses

Microsoft Excel (version 2018) was used to aggregate data from the study protocols. IBM SPSS Statistics (version 28.0.0.0) was used for statistical analyses. The independent-samples Mann-Whitney test was used for comparisons of age of onset in PFAPA with vs. without atypical features and girls vs. boys. The chi-square test was used for comparison of the prevalence of atypical features, while Fisher’s exact test was used to compare the response to treatment in different age groups and in patients with vs. without atypical features. The related-samples Wilcoxon signed rank test was used to compare laboratory test results during and between fever episodes. *P*-values of < 0.05 were considered significant.

Mean annual incidence rates were estimated for PFAPA patients aged 0–17 years at disease onset and separately for patients with disease onset before 5 years of age. The number of PFAPA cases were used as the numerators, while the population in the distinct age groups during the study period were used as the denominators.

## Results

**Fig. 3:**
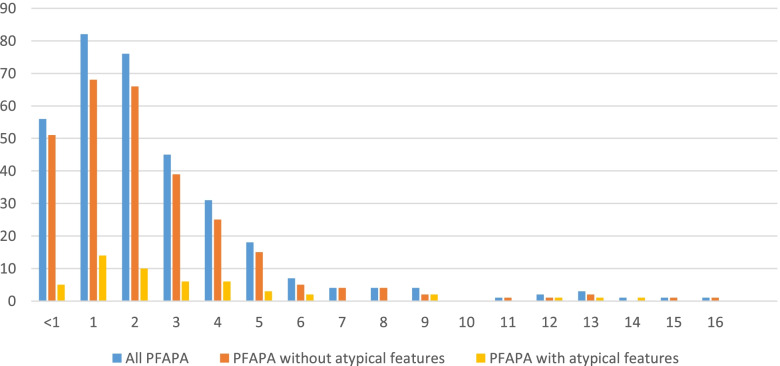
Distribution of age of onset of PFAPA

In our study, a total of 336 patients diagnosed with PFAPA were identified. They included 291 patients with symptom onset before the age of 5 years and 45 patients with later onset. The median age of onset was 2 years (range 0.1–16 years). The distribution of age of onset is shown in Fig. 3. Of the 336 patients, 156 (46%, 95% CI [41, 51]) were girls and 180 (54%, 95% CI [49, 59]) were boys. Atypical features were found in 49 children. There was no significant difference in age of onset between girls vs. boys (*p* = 0.452) or patients with vs. without atypical features (*p* = 0.197).

### Clinical characteristics

According to the criteria used, all the patients had at least one of the associated symptoms of aphthous stomatitis, pharyngitis, or cervical adenitis, of which pharyngitis was the most common. Additional symptoms such as abdominal pain and myalgia/arthralgia were also common. The characteristics of fever episodes, frequency of different symptoms and a positive family history of recurrent fevers can be found in Table 2. In 85% of the patients in the cohort, the clinical diagnostic workup had been performed by one of the authors of this article (PW, SB, JH), while the remaining children were assessed by other pediatricians at our clinics.

**Table 2:** Characteristics of fever episodes, family history and frequency of different symptoms during fever episodes

	All PFAPA patients (*n* = 336)	PFAPA without atypical symptoms (*n* = 287)		PFAPA with atypical symptoms (*n* = 49)	
		< 5 years (*n* = 252)	≥ 5 years (*n* = 35)	< 5 years (*n* = 39)	≥ 5 years (*n* = 10)
Age of onset, median (range)	2.0 (0.1–16)	2.0 (0.1–4.9)	6.5 (5–16)	1.9 (0.1–4.5)	9.2 (5–14)
Distribution females/males	46% / 54%	47% / 53%	46% / 54%	44% / 56%	40% / 60%
Length (days) of fever episodes, median (range)	4.5 (1.5–9)	4.5 (1.5–6.5)	3.8 (1.5–5.5)	4.5 (2.5–9)	5 (2.5–7)
Interval (days) between episodes, median (range) ^a^	28 (11–84)	28 (11–70)	28 (14–84)	28 (14–56)	28 (16–31)
Family history					
Recurrent fevers	28%	27%	34%	28%	30%
Recurrent fevers in 1st degree relative	20%	20%	23%	18%	10%
Tonsillectomy	25%	25%	26%	21%	50%
Tonsillectomy in 1st degree relative	13%	14%	3%	15%	20%
Pharyngitis^b^	94%	95%	100%	95%	60%
Aphthous stomatitis	43%	44%	34%	46%	60%
Cervical adenitis	81%	81%	71%	90%	80%
Gastrointestinal (GI) symptoms					
Any GI symptom, excl diarrhea	46%	44%	40%	54%	50%
Abdominal pain	33%	33%	31%	36%	30%
Nausea/vomiting	17%	17%	11%	23%	20%
Myalgia/arthralgia	33%	31%	34%	36%	70%
Headache	15%	13%	23%	18%	30%
Neurological symptoms					
Dizziness	1%	0.4%	3%	3%	0
Febrile seizures	3%	4%	0	3%	0

^a^As stipulated in the PFAPA syndrome criteria, all patients had regularly recurring fevers during some stage of their illness but many also had periods with less regular episodes. The interval between episodes represents the period with regularly recurring fevers

^b^The term pharyngitis was used in the Modified Marshall criteria and therefore also used here. In this study, this includes all patients with a record of inflammation in the throat, regardless of whether tonsils were involved or not

The most common atypical symptom was skin rash (documented in 4.8% of patients), followed by cold- or exercise-induced symptoms (documented in 3.6% of patients) and long episodes (documented in 3.6% of patients). Only 8 patients had more than one atypical symptom. The presence of atypical features in the different age groups is presented in Table 3. There was no significant difference in the occurrence of any atypical features between patients with onset before 5 versus after 5 years of age (*p* = 0.12). The prevalence of each atypical feature was low in both age groups and appears to be fairly evenly distributed between age groups; further statistical subgroup analyses were not considered meaningful due to few patients in the subgroups.

**Table 3:** Atypical symptoms in PFAPA with age of onset < 5 vs ≥ 5 years

	All PFAPA (*n* = 336)	Onset < 5 years (*n* = 291)	Onset ≥ 5 years (*n* = 45)
Any atypical symptom	49/336 (14.6%)	39/291 (13.4%)	10/45 (22.2%)
Skin rash	16/336 (4.8%)	14/291 (4.8%)	2/45 (4.4%)
Cold- or exercise-induced symptoms	12/336 (3.6%)	8/291 (2.7%)	4/45 (8.9%)
Episodes lasting ≥7 days	12/336 (3.6%)	10/291 3.4%)	2/45 (4.4%)
Diarrhea	6/336 (1.8%)	4/291 (1.4%)	2/45 (4.4%)
Chest pain	4/336 (1.2%)	2/291 (0.7%)	2/45 (4.4%)
Conjunctivitis	3/336 (0.9%)	3/291 (1.0%)	0
Arthritis	2/336 (0.6%)	2/291 (0.7%)	0
Periorbital edema	2/336 (0.6%)	2/291 (0.7%)	0
Severe abdominal pain	1/336 (0.3%)	1/291 (0.3%)	0
Sensorineural hearing loss	1/336 (0.3%)	1/291 (0.3%)	0
More than one atypical symptom	8/336 (2.4%)	6/291 (2.1%)	2/49 (4.4%)

Results of genetic tests for monogenic periodic fever syndromes were found in the medical records of 16 patients. Genetic testing was performed more often in patients with origin in the Eastern Mediterranean area (8/16) and in patients with atypical features (10/16). Variants in *MEFV*, the gene causing familial Mediterranean fever, was found in 5 patients (two heterozygous E148Q, one heterozygous R202Q, one heterozygous V726A and one compound heterozygous M680I + R202Q). One patient had a polymorphism F402L in *NLRP12*. None of these genetic variants were considered to be disease causing, although we cannot exclude that they may modify the phenotype.

**Fig. 4:**
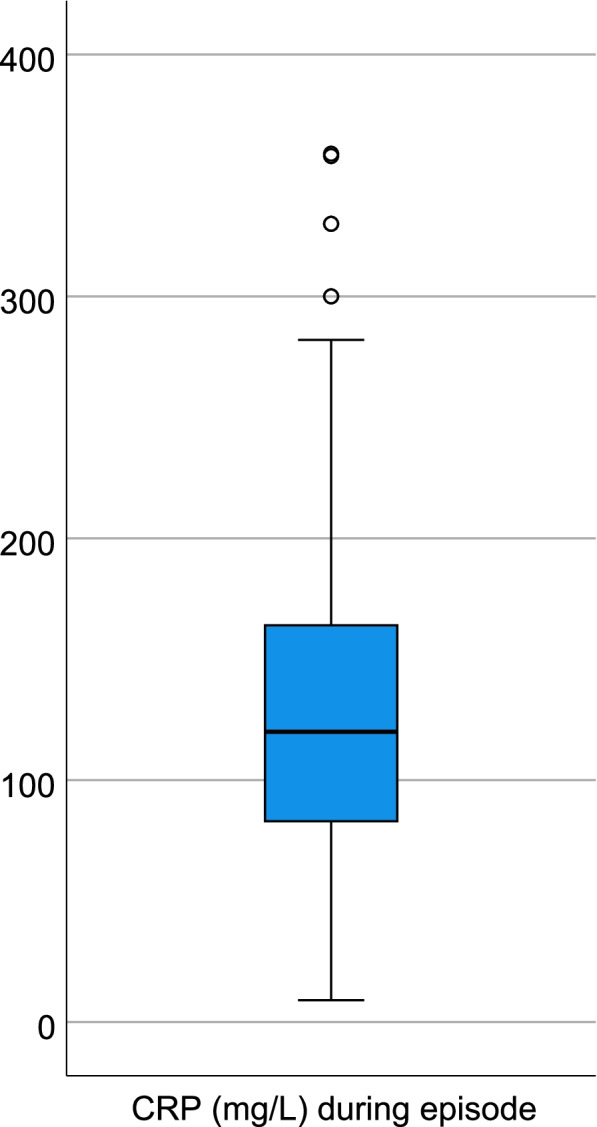
Distribution of CRP levels during fever episodes

Laboratory test results were retrieved from the medical records when available. The results of the main laboratory investigations are shown in Table 4 and Fig. 4. Inflammatory markers (C-reactive protein (CRP), serum amyloid A (SAA) and/or erythrocyte sedimentation rate (ESR)) were available for 94% of the patients during fever episodes and 79% in asymptomatic intervals. SAA was measured in 161 patients during febrile episodes and in 197 patients in the afebrile interval. In febrile patients, SAA was > 300 mg/L (the upper limit of detection) in 156/161 (range 73- > 300). In the afebrile interval, SAA was < 11 mg/L (the lower limit of detection) in 164/197 patients (range < 11–66).

**Table 4:** Median laboratory test results (range) during and between fever episodes

	All PFAPA, *n* = 336		
	During fever episode	Asymptomatic interval	Statistical significance
CRP (mg/L)^a^	120 (9–359) *n* = 315	0 (0–17) *n* = 232	*p* < 0.0005 (208 paired samples)
Leukocyte count (× 10^9^/L)	13.6 (4.1–32.8) *n* = 234	7.6 (3.1–16) *n* = 236	*p* < 0.0005 (176 paired samples)
Lymphocyte count (×10^9^/L)	2.8 (0.5–14) *n* = 189	3.2 (0.83–10.1) *n* = 211	*p* < 0.001 (133 paired samples)
Neutrophil count (× 10^9^/L)	9.2 (1.8–22.4) *n* = 203	3.4 (1.1–9.6) *n* = 214	*p* < 0.0005 (145 paired samples)
Monocyte count (×10^9^/L)	1.0 (0–3.8) *n* = 185	0.4 (0.13–1.44) *n* = 211	*p* < 0.0005 (130 paired samples)
Eosinophil count (×10^9^/L)	0.1 (0–1.1) *n* = 179	0.2 (0.02–2.1) *n* = 209	*p* < 0.001 (126 paired samples)
Hemoglobin (g/L)	114 (93–170) *n* = 219	123 (98–165) *n* = 230	*p* < 0.001 (163 paired samples)
Thrombocyte count (×10^9^/L)	279 (115–570) *n* = 206	349 (167–812) *n* = 228	*p* < 0.0005 (155 paired samples)

^a^When CRP was denoted as > 180 vs < 5 in the medical records, it was transformed to the numerical 180 vs 0 respectively

### Treatment

Antipyretic treatment (acetaminophen and/or ibuprofen) was documented in 115/336 patients (34%) but was undoubtedly underreported in the medical records, as these drugs are usually purchased over the counter without a prescription. 158/336 patients (47%) were prescribed corticosteroids (betamethasone) to abort single febrile episodes. Patients were advised to use the drug only on special occasions, limiting the number of doses to 2–3 per year due to concern relating to side-effects and the shortening of the interval between episodes. This reflects the clinical practice in our clinics [23]. The effect of betamethasone was documented in 81 patients, of whom 73 (90%) reported a total alleviation of symptoms, 8 reported a partial response and none reported it as completely ineffective. Of the patients with a partial response, 5 reported that the fever was lowered but the child was still not feeling well and 3 reported that the symptoms disappeared quickly but reappeared within a few hours. The effect was similar in patients with vs. without atypical features. In the group without atypical features, follow-up data were available for 69 patients, of whom 61 (88%) reported a complete response and 8 (12%) a partial response. In the group with atypical features, all 12 patients for whom follow-up was documented reported a complete response (non-significant, *p* = 0.59). In patients in whom corticosteroids were documented to mitigate fever, 25% reported a shortening of fever-free intervals. Continuous treatment was rare in the cohort and colchicine was the only prescription drug used, given to 7/336 patients (2%). Of these, 3 patients had atypical features. Five patients reported fewer and less severe episodes after starting colchicine, one patient reported no effect and one patient was lost to follow-up. Tonsillectomy was performed on 116/336 patients (35%) of patients. Data on outcome after tonsillectomy were available for 70 patients, of whom 65 (93%) reported a complete or partial resolution of symptoms. However, 6 of them returned to our clinic later with a relapse of symptoms. The remaining 5 patients reported no improvement after tonsillectomy. Data on the outcome of tonsillectomy in the different subgroups can be found in Table 5. The outcome of tonsillectomy did not differ significantly between patients with vs. without atypical features (*p* = 0.47) or in patients with symptom onset before vs. after 5 years of age (*p* = 0.43).

**Table 5:** Outcome of tonsillectomy in PFAPA with vs. without atypical symptoms

	All PFAPA	PFAPA without atypical symptoms	PFAPA with atypical symptoms
No of pts. with tonsillectomy	116/336 (34.5%)	96/287 (33.4%)	20/49 (40.8%)
Follow-up available^a^	70/116 (60.3%)	55/96 (57.3%)	15/20 (75%)
Complete resolution of symptoms reported	37/70 (52.9%)	31/55 (56.4%)	6/15 (40%)
Improved but not completely asymptomatic	22/70 (31.4%)	16/55 (29.1%)	6/15 (40%)
Resolution of symptoms after surgery but relapse of fever episodes later	6/70 (8.6%)	5/55 (9.1%)	1/15 (6.7%)
No improvement	5/70 (7.1%)	3/55 (5.5%)	2/15 (13.3%)

^a^All patients were encouraged to contact their doctors if they did not improve after surgery. As a result, it can be assumed that a large percentage of those that have not been followed post-surgery improved after the procedure

### Incidence

For the calculation of incidence, 251 patients with PFAPA, symptom onset between January 1, 2006, and December 31, 2016, and resident in the catchment area were identified. Patients were included in the incidence cohort regardless of whether they had atypical features or not. 221 of the patients were < 5 years of age at the onset of the PFAPA episodes while 30 were 5 years or older. When including all the patients with PFAPA regardless of age at symptom onset, the mean annual incidence was estimated at 0.86/10,000 children < 18 years of age. In the group with symptom onset before 5 years of age, the mean annual incidence was estimated at 2.6/10,000 children < 5 years of age.

## Discussion

This study describes one of the largest cohorts of PFAPA patients to date, derived from a restricted geographical area in western Sweden with a well-defined background population. The mean annual incidence of PFAPA in the young age group (2.6/10,000 children < 5 years of age) estimated in this study is comparable to that previously reported in other Nordic countries [18, 20]. Our study also suggests an annual incidence of PFAPA of 0.86/10,000 in children up to 18 years of age. This is ten times the incidence reported by Ng et al. [21]. One explanation of this could be that their cohort was limited to patients seen at a tertiary hospital, while our study includes pediatric facilities at secondary level.

In this study, we also present the age distribution of PFAPA onset, showing that almost 90% of children with PFAPA experience their first symptoms before the age of 5 years and fewer than 3% present at ages above 10 years. The distribution of age of onset was the same regardless of whether or not atypical features were present. Even though this study is limited to children < 18 years of age, the rare presentation of PFAPA syndrome in teenagers might be indicative of the situation in adults as well, but this requires further studies. It is interesting to note that, even in the first description of the syndrome by Marshall et al. in 1987 [1], most but not all patients experienced their first symptoms before 5 years of age and that, despite accumulating cases with later onset, the syndrome’s preponderance in low ages is clear. The way the immunologic mechanisms of PFAPA relate to the maturing immune system of young children remains an exciting research topic.

Many of the clinical findings in this study are in accordance with previous studies (Table 6). In this study, we define subgroups of PFAPA based on the presence or absence of atypical features. Most patients in our cohort did not have atypical features according to our definition and each atypical feature was only found at low rates. Twelve patients in our cohort reported that cold or exercise could induce fever episodes, which to our knowledge has not been described in PFAPA patients before. As this might seem contradictory to the regular recurrence of fever episodes in PFAPA syndrome, it is worth pointing out that this feature was mostly described late in the disease course where episodes were less regular and sometimes in patients with a relapse of fever episodes after tonsillectomy. Patients with and without atypical features were similar regarding age of onset, sex distribution and characteristics of the fever episodes, as well as in their response to treatment with betamethasone and tonsillectomy.

**Table 6:** Characteristics of PFAPA patients and frequency of symptoms during fever episodes in different studies

Authors	Year	N	Sex M/F %	Age of onset^a^ (years)	Time between episodes^a^ (weeks)	Duration of episodes^a^ (days)	Pharyngitis (%)	Cervical adenitis (%)	Aphthous stomatitis (%)	Abdominal pain (%)	Diarrhea (%)	Skin rash (%)	Conjunctivitis (%)
Marshall et al. [1]	1987	12	58/42	N/A	4.5	5	75	67	75	50	N/A	8	N/A
Thomas et al. [9]	1999	94	55/45	2.8	4	4.8	65	77	67	45	30	15	N/A
Padeh et al. [24]	1999	28	71/29	4.2	5.1	4.3	100	100	68	18	N/A	N/A	N/A
Tasher et al. [25]	2006	54	61/39	1.9	3.7	5.3	96	61	39	65	13	4	N/A
Feder et al. [26]	2010	105	62/38	3.3	4.3	4.1	85	62	38	41	N/A	N/A	N/A
Forsvoll et al. [18]	2013	46	70/30	0.9	3.5	4	approx. 80	approx. 90	approx. 50	approx. 40	N/A	N/A	N/A
Krol et al. [27]	2013	125	50/50	1.9	4	3.5	91	78	41	23	5	3	3
Hofer et al. [28]	2014	301	53/47	1.7	4	4	90	78	57	45	16	13	5
Perko et al. [29]	2015	81	63/37	2.1	4	4.2	98	94	56	51	22	12	N/A
Batu et al. [30]	2016	131	65/35	1.8	3.7	4	96	53	43	46	11	5	N/A
Amarilyo et al. [31]	2020	303	60/40	3.0	4.7/3.5/3.9 ^b^	4.8/4.3/3.9 ^b^	88/96/90 ^b^	47/51/47 ^b^	29/38/33 ^b^	24/55/64 ^b^	N/A	N/A	N/A
Results in this study	2022	336	54/46	2.0	4	4.5	94	81	43	33	2	5	1

^a^Mean or median, depending on what was reported

^b^This study investigates differences between ethnic groups and the numbers refer to the three reported groups Non-Mediterranean/Mediterranean/Multiethnic

Both CRP and SAA were commonly used inflammatory markers in our cohort and both were distinctly elevated during episodes and normalized in the fever-free intervals in most patients, as has been shown previously [4, 27, 32, 33]. In a few patients we could only identify marginally elevated CRP during fever episodes. Due to the retrospective design, it is difficult to determine the significance of this. It could be that the test was taken too early during the febrile episode, but it might also be that not all patients who fulfill the diagnostic criteria of PFAPA have very high CRP levels during the entire course of the disease. In our experience, this applies particularly to older children and patients who are experiencing milder episodes after several years of periodic fevers or after tonsillectomy. Further, prospective studies are needed to gain more insight on this. SAA is a sensitive inflammatory marker and is commonly used in the evaluation of patients with autoinflammatory disorders as continuous elevation is a sign of subclinical inflammation that may lead to secondary amyloidosis [34]. The normalization of SAA between episodes can be useful in differentiating PFAPA from other autoinflammatory conditions and it is also reassuring in the context of secondary amyloidosis, which has not been reported in patients with PFAPA [35]. In our study, 33 patients had slightly elevated SAA values even in the interfebrile interval. Again, it is difficult to determine the significance of this due to the retrospective design and it could be that the test was taken too close to a febrile episode to have completely normalized. Compared with the interfebrile intervals, febrile episodes were associated with increased levels of neutrophils and monocytes in the blood, while lymphocyte and eosinophil counts decreased. Hemoglobin levels decreased during febrile episodes and thrombocytosis was seen in the afebrile period, reflecting the inflammatory process. This confirms what has previously been found in smaller cohorts [5, 33].

The best way to treat patients with PFAPA is still a somewhat controversial issue. In this cohort, tonsillectomy was performed in a third of the patients. Unfortunately, follow-up data were not available for all patients, but more than 90% of patients with available data after tonsillectomy reported a complete or partial resolution of symptoms after the procedure. This compares with the findings in a recently conducted review that found surgery curative in 92% of patients [36]. The outcome of tonsillectomy was similar in different subgroups in our study, regardless of age at onset and whether or not atypical features were present. A few patients do not improve after tonsillectomy and, even in those who benefit in the short term, symptoms can return after a prolonged period of time. Families should be carefully informed of the potential risks and benefits of the procedure and a shared decision-making approach should be used. The use of corticosteroids to abort febrile episodes in PFAPA is a well-established practice. Although few side-effects have been reported, we advise our patients to use it with discernment in order to avoid potential side effects on growth and bone mineralization [3, 23]. We choose to give our patients betamethasone rather than prednisolone as the prednisolone oral solution is not available in Sweden, and because of a possible advantage of the longer duration of action of betamethasone. Maintenance treatment with colchicine was rare in this retrospective cohort but has probably increased in recent years as more data on its effectiveness have emerged [37, 38]. Future studies are needed to determine the best treatment regime for PFAPA.

### Methodological considerations and limitations to the study

Determining the incidence of PFAPA is a challenging task in several ways. Awareness of the syndrome has been and might still be limited among patients, parents and physicians, resulting in the probable under-diagnosis of the syndrome and, as a result, the under-estimation of the incidence in a study relying on the number of diagnosed patients. The retrospective approach in this study, building on the identification of PFAPA patients by searching for ICD-10 codes, also represents a limitation, as there is no specific ICD-10 code for this condition. The pediatric referral centers included in this study work in close collaboration with one another and have worked systematically to raise awareness of periodic fevers since the beginning of the new millennium. We have an established routine of using ICD-10 code D898 for PFAPA. With the addition of a review of medical records for patients with the ICD-10 symptom code for fever (R509), we believe that essentially all the patients diagnosed with PFAPA in our hospitals were identified. We might nevertheless have missed some patients that were diagnosed and treated by pediatricians in primary care, ENT specialists or even GPs and never referred to the pediatric departments in the hospitals in the catchment area. According to local routines and our broad network in the area, this is seldom the case and should not constitute a number of patients large enough to alter the estimated incidence in a significant way.

The retrospective approach of this study limits the data available to what was registered in the medical records and thus represent a minimum occurrence, as all symptoms may not have been documented. However, almost all the patients in this cohort were seen by an experienced pediatrician with expert knowledge of autoinflammatory disorders, using a systematic approach to collecting relevant information regarding signs and symptoms. The agreement of our data with that in previous studies regarding the clinical features supports the proposition that the patients included in our study are representative of the PFAPA syndrome.

The current lack of validated diagnostic criteria for PFAPA is an obstacle to creating homogeneous patient cohorts in the research context. As the modified Marshall criteria do not on their own either exclude other diagnoses or include all patients recognized by most physicians in the field as PFAPA, many researchers define their own criteria for the condition to ensure their studies enroll a well-defined group of patients [5, 26, 28, 39]. In the clinical context, physicians also have diverse perspectives of the signs and symptoms that are required to make a PFAPA diagnosis [40]. The recently proposed classification criteria for PFAPA by Gattorno et al. offers a new approach to the definition of PFAPA patients using positive (presence of) and negative (absence of) clinical variables as well as prerequisites of markedly elevated inflammatory markers, careful consideration of confounding diseases and at least 6 months of disease activity [10]. As they were developed for research purposes, the criteria are explicitly not meant to be employed as diagnostic criteria but may nevertheless be helpful in clinical practice. Renko et al. have suggested wider diagnostic criteria, including patients without evidence of aphthous stomatitis, pharyngitis and cervical adenitis, at least in the context of a population where monogenic autoinflammatory disorders as well as cyclic neutropenia are exceedingly rare, such as in populations with mainly Scandinavian ethnicity [20]. In this study, we chose to define PFAPA according to the modified Marshall criteria but divided the cohort into two different subgroups based on the absence or presence of atypical features. Although atypical features should prompt consideration of other causes of the symptoms, a diagnosis of a monogenic autoinflammatory disorder is uncommon, even in patients who display these features [17], and the classification criteria formulated by Gattorno et al. allow for one atypical feature if the patient fulfils all the other criteria [10]. We considered using these criteria in this study, but the retrospective design made them less appropriate. In addition, they were published after the patients in this cohort were assessed clinically. No significant differences regarding age of onset, sex distribution, clinical features or response to treatment were found in PFAPA patients with vs. without atypical features in this study. Until there is further knowledge of the pathogenesis of PFAPA and specific diagnostic markers have been developed, patients must be evaluated individually, differential diagnosis pursued to a reasonable extent and the PFAPA diagnosis relies on the clinician putting together all the pieces of the puzzle. Expert opinion, which we used for ambiguous cases, has even been suggested as the gold standard for diagnosing PFAPA [28]. We cannot completely exclude the possibility that a few patients with atypical features in this study could have a mAID, but, as the incidence of these disorders is very low in our population, it should not alter the results in any significant way [41].

In addition to the need to segregate PFAPA syndrome from mAID, it must also be distinguished from the more heterogenous group of patients with recurrent fever who do not fulfill the clinical or genetic criteria for any defined AID. This group is often referred to as undefined/undifferentiated AID (uAID) or syndromes of undifferentiated recurrent fever (SURF). As the diagnostic criteria for PFAPA are still a subject of debate, it is not clear which patients with PFAPA-like disease but with a not entirely typical presentation should be regarded as PFAPA and which should be included in the more ambiguous group of uAID/SURF [42–46]. Our study shows that PFAPA patients who fulfill the modified Marshall criteria and display additional atypical features are similar to patients without atypical features regarding age of onset, sex and response to treatment. While our study confirms that the age of onset of PFAPA is < 5 years of age in most patients, this is not an absolute limit and patients with onset in older ages should also be classified as PFAPA if they fulfil the other criteria. We propose that patients who do not have the key features of regularity and signs and symptoms from the oro-pharyngeal area should be designated SURF. Further studies need to be conducted to investigate whether these patients are different from PFAPA patients regarding the prognosis and outcome of treatment.

## Conclusions

This study adds to the understanding of the epidemiology of PFAPA syndrome by presenting incidence rates based on a large number of patients in different age groups in a population-based setting. The annual incidence of 2.6/10,000 of PFAPA in children aged < 5 years in this study is strikingly similar to the incidence rates in two previously published papers [18, 20]. This study also shows that the onset of PFAPA is much more common in young children, with a peak in 1-year-olds, and then appears to wane in older ages, which is also reflected in the lower incidence rate of 0.86/10,000 in ages 0–17 years. Signs and symptoms of PFAPA syndrome are similar in children with symptom onset before and after 5 years of age in this study.

## Data Availability

The datasets used and/or analyzed during the current study are available from the corresponding author on reasonable request.

## Abbreviations

PFAPA: Periodic fever, aphthous stomatitis, pharyngitis and cervical adenitis
mAID: Monogenic autoinflammatory disorders
CRP: C-reactive protein
SAA: Serum amyloid A
ESR: Erythrocyte sedimentation rate
uAID: Undefined/undifferentiated AID
SURF: Syndromes of undifferentiated recurrent fever
